# Around the Hospitals

**Published:** 1903-10-24

**Authors:** 


### AROUND THE HOSPITALS.

### THE ITALIAN HOSPITAL.

The secretary of the Italian Hospital complains that in our notice of his institution under the above heading we stated that no medical and surgical report accompanied the annual report of the hospital. Our representative was led into making this comment owing to the fact that no report of cases was included in the annual report handed to the representatives of the press at the annual meeting. We find that in the full report which was issued later the medical and surgical reports are included.

